# Multiple Myeloma Associated Chronic Inflammatory Demyelinating Polyradiculoneuropathy: The Importance of Continued Surveillance

**DOI:** 10.7759/cureus.899

**Published:** 2016-11-28

**Authors:** Adebayo A Fasanya, Michael F Loncharich, Viral Gandhi, Sandeep Rana, Marvin Balaan

**Affiliations:** 1 Pulmonary and Critical Care Medicine, Allegheny General Hospital; 2 Medicine, Temple University School of Medicine; 3 Neurology, Allegheny General Hospital

**Keywords:** cidp, neuropathy, weakness, paraproteinemia

## Abstract

Chronic inflammatory demyelinating polyradiculoneuropathy (CIDP) is an autoimmune disease presenting with weakness and numbness in a remitting or chronic progressive course. It is known to have several clinical presentations and several associated diseases. CIDP has been associated with multiple myeloma, monoclonal gammopathy of undetermined significance (MGUS), and other paraproteinemias. We present a case of refractory CIDP in which the initial workup for multiple myeloma was negative, and multiple myeloma was then diagnosed two and half years later. Treatment of the multiple myeloma led to clinical improvement. This case is instructive in that perhaps more frequent surveillance for paraproteinemia in patients with CIPD, even after a negative initial workup, could lead to a better clinical outcome.

## Introduction

Chronic inflammatory demyelinating polyradiculoneuropathy (CIDP) is a peripheral neuropathy characterized by distal and proximal sensorimotor deficits. Several monoclonal gammopathies and lymphoproliferative disorders have been associated with CIDP [1]. 

Multiple myeloma has been associated with more than one neuropathy. Axonal, axonal-demyelinating, and other demyelinating patterns have been described [2]. The most common presentation of neuropathy in multiple myeloma is a mild, symmetric sensorimotor loss with sensory losses greater than motor losses and with distal involvement greater than proximal. Despite this variable presentation, several trials found idiopathic CIDP to be indistinguishable from CIDP related to monoclonal gammopathies [2-3]. 

Monoclonal gammopathies are more prevalent in peripheral neuropathies of an unknown cause than in the general population (10% vs 1 - 3%) [4]. One-third of patients diagnosed with multiple myeloma show clinical signs of neuropathy at the time of diagnosis [5]. Subclinical neuropathy can be detected in over 50% of multiple myeloma cases [4].

The following case describes a 47-year-old male who presented with CIDP and was found to have hypercalcemia. Workup for plasma cell dyscrasias and amyloidosis was negative at that time. Two years later, he was found to have lytic lesions of the bony pelvis, triggering a workup for multiple myeloma. This workup resulted in a diagnosis of multiple myeloma. It is important to consider paraproteinemias as contributing or causative comorbidities early in the course of CIDP.

Informed patient consent was obtained. 

## Case presentation

A 47-year-old male presented with bilateral upper and lower extremity weakness, worse in the distal muscles, with sensory loss. Cerebrospinal fluid analysis showed albuminocytologic dissociation. An electromyography and nerve conduction study was consistent with acquired demyelinating sensory and motor polyneuropathy. On routine labs, he was found to be hypercalcemic (10.8 mg/dL), so he was worked up for amyloidosis vs monoclonal gammopathy of undetermined significance (MGUS) vs multiple myeloma. Serum protein electrophoresis (SPEP), immunofixation, and fat pad biopsy were all negative. Bone marrow biopsy was inconclusive due to an inadequate sample. He had no further workup at that time and was treated with IV immunoglobulin (IVIG) and prednisone. 

Over the course of the following year, he had several relapses of worsening weakness that required IVIG therapy, increasing doses of oral steroids, and inpatient rehabilitation. During this time, he suffered numerous infections, including pneumonia, methicillin-sensitive Staphylococcus aureus (MSSA) sepsis, Fournier gangrene, C. difficile infection, and cervical epidural abscess. His functional status declined such that he had spent 50 weeks in hospitals, inpatient rehab, and long-term acute care facilities. 

Two years after his initial diagnosis with CIDP, the patient presented with an acute change in mental status and hypercalcemia. He had a tracheostomy placed one month prior to admission for chronic hypoxic respiratory failure following pneumonia. On exam, he had fatigable ptosis and complex ophthalmoparesis with bilateral abduction and adduction weakness, concerning for bilateral internuclear ophthalmoplegia (INO). His cranial nerves were otherwise intact. Muscle strength was 0/5 throughout his lower extremities, except 1/5 on plantar flexion. Upper extremity strength was 3/5 throughout. The sensory exam revealed absent sensation in all four distal extremities and normal sensation in the upper extremities, trunk, and face. Reflexes were 2/4 at the Achilles' tendon and absent elsewhere. Coordination and gait could not be tested due to disability.

**Figure 1:**
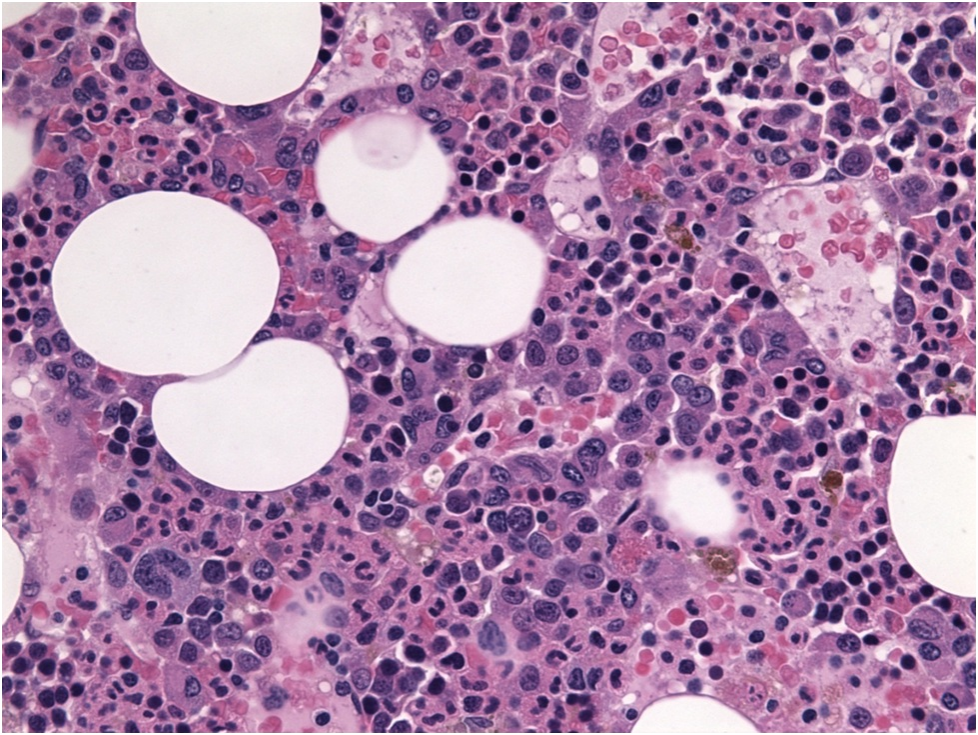
H and E stain showing atypical plasma cells with coarse chromatin, nuclear irregularities and multinucleated forms

**Figure 2:**
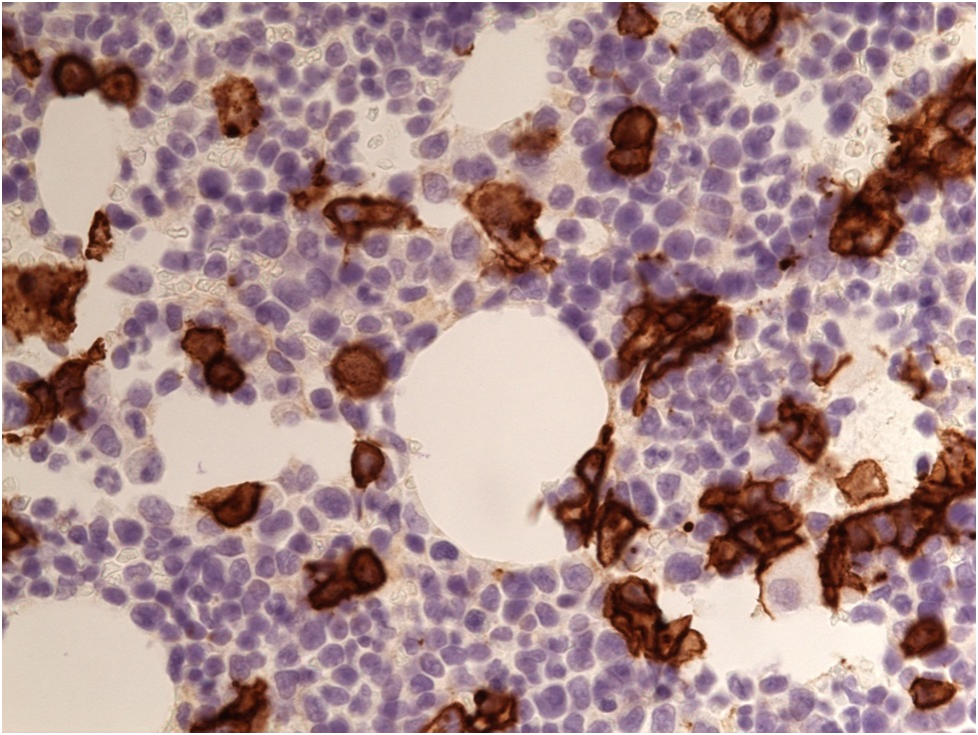
CD 138 identifying malignant plasma cells in bone marrow

On initial workup, he was again found to be hypercalcemic (13.8 mg/dL). A CT-spine was done to evaluate his pain and revealed several small, lytic lesions scattered in the bony pelvis. He was worked up for multiple myeloma vs bony metastases. Urine protein electrophoresis showed an IgG spike (4,106 mg/dL) and elevated random urine protein (14.7 mg/dL). Serum protein electrophoresis showed hypoalbuminemia (2.3 mg/dL) with an M-spike (γ-globulin 3.2 g/dL, M-spike 10.8 g/dL). Immunofixation revealed IgG kappa monoclonal gammopathy (IgG 3,427 mg/dL, free kappa light chains 8.13 mg/L, kappa/lambda ratio 3.73). A bone marrow biopsy was consistent with multiple myeloma (Figures 1-2).

 

Skeletal survey revealed a permeative pattern worrisome for marrow replacement in the pelvis, humerus, and femur. There was no evidence of bony metastases on computerized tomography, and free and total prostate-specific antigen (PSA) were within normal limits.

CIDP was treated with dexamethasone, 20 mg weekly, and IVIG on a three-week cycle, weekly for two weeks, then daily for five doses in the third week. His multiple myeloma was treated with carfilzomib, cyclophosphamide, and dexamethasone on a 28-day cycle. At the time of this writing, the patient has received his first two doses. At this early stage, minor improvements have already been noted in his muscular strength. His quadriceps strength improved from 0/5 to 1/5 and plantar and dorsiflexion strength improved to 2/5. 

## Discussion

This patient was found to have multiple myeloma with an IgG monoclonal gammopathy. Seventy-five percent of IgG gammopathies do not have an associated neuropathy [4]. Some cases have antibodies against neural antigens without clinical neuropathy [6]. At initial diagnosis, approximately 33% of multiple myeloma patients have a peripheral neuropathy [5]. When investigated from the opposite perspective, 10% of patients with cryptogenic neuropathies are found to have a preexisting paraproteinemia [7].

While IgG is the most common monoclonal gammopathy, IgM accounts for more than 50% of paraprotein-associated demyelinating neuropathies [4]. This raises the question of causality. Is multiple myeloma with an IgG paraproteinemia a causal or comorbid condition with CIDP? There are cases in which perineural or perivascular IgG kappa deposits were found [8] and IgG deposition in myelin sheaths and widening of the lamellae have been described on electron micrograph (EM) [8]. Not all cases have such strong supporting evidence of causality, and nerve biopsy is often not performed, as in this case. There are cases in which patients have antibodies against neural antigens with other evidence of neuropathy [6]. 

In the case presented here, CIDP was diagnosed two years before multiple myeloma. Rison, et al. found that neural symptoms may precede other clinical manifestations of paraproteinemias by years [7]. There have been reports of delayed diagnosis of MGUS in CIDP patients [3]. This patient’s pattern of sensory losses greater than motor losses and bilateral INO are patterns of neuropathy that have been described in multiple myeloma-associated CIDP [9]. Further, his initial poor response to IVIG is a pattern described in other monoclonal gammopathy-associated neuropathies [1].

Given that paraproteinemia-neuropathies may be indistinguishable from CIDP, how should they be worked up? Protein electrophoresis and immunofixation of the serum and the urine are routinely used [7]. Some have suggested that nerve biopsy may elucidate the neuropathy’s etiology, but the result is not consistent [9]. Ultrasound has been proposed as a less invasive way to diagnose paraproteinemic neuropathies. Peripheral nerve ultrasound can be used in conjunction with nerve conduction studies to differentiate anti-myelin-associated glycoprotein, CIDP, and M-protein-associated neuropathies [10]; however, this does not help in multiple myeloma due to its variable neurotoxic patterns. 

This patient was initially treated with mycophenolate mofetil and rituximab, which failed to halt disease progression. Treatment with IVIG was more successful at slowing disease progression but has not prevented relapses. We are hopeful that treating his newly diagnosed multiple myeloma will lead to clinical improvement. At the time of this writing, the patient has only received two doses of his new chemotherapy regimen but already shows improvements in distal muscle strength as described above.

## Conclusions

CIDP has been associated with multiple myeloma, MGUS, and other paraproteinemias. Monoclonal gammopathy-associated CIDP progresses more rapidly and is more likely to be refractory to therapy [5]. Clinically, this association is important to evaluate as it affects the natural progression of disease. Perhaps in this case, yearly or more frequent workup for multiple myeloma might have made the diagnosis earlier and resulted in a better outcome. 
